# Enhanced lipid production by addition of malic acid in fermentation of recombinant *Mucor circinelloides* Mc-MT-2

**DOI:** 10.1038/s41598-021-92324-7

**Published:** 2021-06-16

**Authors:** Yao Zhang, Qing Liu, Pengcheng Li, Yanxia Wang, Shaoqi Li, Meng Gao, Yuanda Song

**Affiliations:** 1 Colin Ratledge Center for Microbial Lipids, School of Agricultural Engineering and Food Science, Shandong University of Technology, 266 Xincun West Road, Zibo, 255000 People’s Republic of China; 2 Key Laboratory of Shandong Provincial Universities for Technologies in Functional Agricultural Products, Shandong University of Technology, 266 Xincun West Road, Zibo, 255000 People’s Republic of China

**Keywords:** Biological techniques, Biotechnology, Microbiology

## Abstract

In our previous work, we reported a novel approach for increasing lipid production in an oleaginous fungus *Mucor circinelloides* by overexpression of mitochondrial malate transporter protein. This transporter plays a vital role in fatty acid biosynthesis during malate and citrate transport systems in oleaginous fungi. In this study, the controlling metabolic supplementation strategy was used to improve the lipid production by overexpression of malate transporter protein in *M. circinelloides* strain coded as Mc-MT-2. The effects of different metabolic intermediates on lipid production in batch fermentation by Mc-MT-2 were investigated. The optimal lipid production was obtained at 0.8% malic acid after 24 h of fermentation. Furthermore, in fed-batch bioreactors containing glucose as a carbon source supplemented with malic acid, the highest cell growth, and lipid production were achieved. The resulting strain showed the fungal dry biomass of 16 g/L, a lipid content of 32%, lipid yield of 5.12 g/L in a controlled bench-top bioreactor, with 1.60-, 1.60- and 2.56-fold improvement, respectively, compared with the batch control without supplementation of malic acid. Our findings revealed that the addition of malic acid during fermentation might play an important role in lipid accumulation in the recombinant *M. circinelloides* Mc-MT-2. This study provides valuable insights for enhanced microbial lipid production through metabolic supplementation strategy in large scale and industrial applications.

## Introduction

The recent energy crisis has triggered significant attention on the microbial synthesis of lipids, which are being considered as potential sources of biofuels^1–5^. Additionally, microbial lipids have been receiving a tremendous amount of attention recently, especially with regard to their high content of polyunsaturated fatty acids (PUFAs) such as arachidonic acid (AA), docosahexaenoic acid (DHA), eicosapentaenoic acid (EPA) and γ-linolenic acid (GLA) that have nutritional benefits^6–8^. Therefore, the scope of potential applications of microbial lipids is much broader and also includes chemical (fuel additives, lubricants), pharmaceutical, cosmetics and medical industries, extending even to the food industry.

In many microorganisms, including bacteria, microalgae, yeast and filamentous fungi, can accumulate more than 20% (w/w) of lipid of their cell dry weight and are defined as oleaginous microorganisms^4,5^. One of the most promising oleaginous microorganisms for lipid production is the zygomycete fungus *Mucor circinelloides*, which is of industrial interest because it can produce high level of a valuable omega-6 polyunsaturated fatty acid GLA^9–11^. Moreover, *M. circinelloides* has emerged as a well-known model of oleaginous fungus because of the availability of genome sequence and well-developed tools for genetic engineering^12–16^.

In order to improve lipid production to meet the requirements of industrial applications, current research has focused on genetic engineering of the oleaginous microorganisms by manipulation of transport pathways or overexpression of genes encoding crucial metabolism enzymes^12–16^. In our previous work, we presented a novel approach for increasing the lipid production in an oleaginous fungus *M. circinelloides* by overexpression of mitochondrial malate transporter protein which plays a vital role in fatty acid biosynthesis during malate and citrate transport systems in oleaginous fungi^13,15,16^. A preliminary study has found that the lipid content of the recombinant strain Mc-MT-2 with overexpression of malate transporter protein was increased by 70% compared to the wild control strain^13^. Thus, the recombinant *M. circinelloides* Mc-MT-2 may be a better candidate for use in lipid production.

Additionally, various cultivation approaches have also been adopted to promote lipid production in oleaginous microorganisms. These approaches include varying the medium composition, cultural condition, nutritional feeding design and utilization of media additives^2,3,9,10,17^. Particularly, the metabolic intermediates such as malic acid, oxaloacetate and pyruvate might be used as a medium supplement for enhancing the metabolic pathways into target product synthesis^18–21^. However, the function and mechanism of supplemented metabolic intermediates on lipid production in oleaginous filamentous fungi is still far from clear.

In the present study, an efficient supplemental metabolite control strategy for recombinant *M. circinelloides* Mc-MT-2 aimed to enhance the lipid production was investigated. Initially, we have attempted to examine the cell growth and lipid production through comparing the medium supplemented with different metabolic intermediates. Subsequently, the supplement of malic acid (including addition concentration and addition time) were also discussed in detail. Furthermore, we developed a fed-batch culture of Mc-MT-2 with glucose feeding strategy and supplementation of malic acid, and finally the lipid yield could reach 5.12 g/L, which was improved 2.56-fold compared with the batch control. These approaches may prove valuable for increasing microbial lipid production through metabolic supplementation strategy in large scale and industrial applications.

## Materials and methods

### Strain and media

The strain used in this study was Mc-MT-2 (overexpression of malate transporter protein), which was previously constructed and stocked in our lab^13^. For seed cultivation, the modified K&R medium consisted of (g/L): glucose or other carbons 30, KH_2_PO_4_ 7.0, MgSO_4_·7H_2_O 1.5, ammonium tartrate 3.3, Na_2_HPO_4_ 2.0, yeast extract 1.5, MnSO_4_·5H_2_O 0.0001, Co(NO_3_)_2_·6H_2_O 0.0001, CaCl_2_·2H_2_O 0.1, CuSO_4_·5H_2_O 0.0001, ZnSO_4_·7H_2_O 0.001, and FeCl_3_·6H_2_O 0.008. The nitrogen-limited basic medium for lipid production is consisting of the following (g/L): glucose or other carbons 80, KH_2_PO_4_ 7.0, MgSO_4_·7H_2_O 1.5, ammonium tartrate 3.3, Na_2_HPO_4_ 2.0, MnSO_4_·5H_2_O 0.0001, Co(NO_3_)_2_·6H_2_O 0.0001, CaCl_2_·2H_2_O 0.1, CuSO_4_·5H_2_O 0.0001, ZnSO_4_·7H_2_O 0.001, and FeCl_3_·6H_2_O 0.008. It should be noted that no matter what carbon source is used, glucose or other intermediates are added according to the constant total amount of carbon. The compound ratio of glucose and other intermediates is 9:1, and the total amount of carbon source remains unchanged. The feeding solutions contained 200 g/L glucose.

All reagents used in cultivations were purchased from Sinopharm Chemical Reagent Co., Ltd (Shanhai, China). All of them were standard commercial products of analytical grade unless otherwise indicated.

### Culture conditions

#### Batch fermentation

100 µL spore suspension (approx. 10^7^ spores/mL) of the recombinant strain Mc-MT-2 was cultivated in 150 mL of K&R medium held in 1 L flask with shaking at 150 rpm for 24 h at 30 °C, and then used at 10% (v/v) to inoculated in a 3 L fermenter (BioFlo 110, New Brunswick Scientific Co., Ltd). Fermenter was controlled at 30 °C with stirring at 500 rpm and aeration at 0.5 v/v min^−1^ for 144 h. The pH was maintained at 6.0 by auto-addition of 4 mol/L NaOH or 2 mol/L HCl. It should be noted that supplemental metabolite control strategy depends on the experimental design. At certain time intervals, samples were collected and analyzed for dry cell weight (DCW) and lipid contents. All experiments were carried out in triplicate.

#### Fed-batch fermentation

The seed culture was started by inoculating 100 µL spore suspension (kept at −80 °C) and cultured for 24 h in 150 mL of K&R medium held in 1 L shake flask at 30 °C and 150 rpm. A 10% (v/v) seed culture was inoculated into nitrogen-limited medium in a 3 L fermenter for fed-batch cultivation. The first one was characterized by batch phase with an initial glucose concentration of 80 g/L. After about 48 h of fermentation, 100 mL feeding solutions (200 g/L glucose) were added every 24 h. Moreover, when fermented for 24 h, 0.8% (w/w) malic acid was added in the fermentation broth. Fermenter was controlled at 30 °C with stirring at 500 rpm and aeration at 0.5 v/v min^−1^ for 120 h. During the whole process, the pH was maintained at 6.0 by auto-addition of 4 mol/L NaOH or 2 mol/L HCl. Antifoam was added manually when necessary. All experiments were carried out in triplicate.

### Analytical methods

The fungi cells were harvested at different fermentation times before filtered, then wash them three times with distilled water. Cells were frozen in ultra-low temperature freezer at −80 °C, followed by freeze drying. The weight of the biomass was determined gravimetrically.

Glucose content in supernatant was measured by glucose oxidase–peroxidase (GOD) using glucose measurement kit (Rongsheng Co.Ltd, China) and the depletion of ammonium was determined by indophenol blue spectrophotometric method^13,22^.

Biomass was collected by filtration and dried by lyophilizer. 20 mg dry weight was taken for cell fatty acids extraction. Pentadecanoic acid (15:0) was added into the freeze-dried cells as an internal standard. The total fatty acids were extracted with chloroform/methanol (2:1, v/v) and methylated with 10% (v/v) methanolic HCl at 60 °C for 3 h. The resultant fatty acid methyl esters were extracted with n-hexane and were analyzed by GC equipped a 30 m × 0.32 mm DB-Waxetr column with 0.25 µm film thickness. The program was as follows: 120 °C for 3 min, ramp to 200 °C at 5 °C per min, ramp to 220 °C at 4 °C per min, hold 2 min^10,22^.

### Statistical analysis

A statistical analysis was carried out using SPSS 16.0 for Windows (SPSS Inc., Chicago, IL). The mean values and the standard error of the mean were calculated from the data obtained from three independent experiments. Student’s* t* test was used to evaluate the differences between the means of the test, and *P* < 0.05 was considered as significantly different.

### Consent to participate

All the authors agreed to participate in the scientific work.

### Consent for publication

All the authors agreed to submit the manuscript.

## Results and discussion

### Effects of supplemented metabolic intermediates on cell growth and lipid production

**Figure 1:**
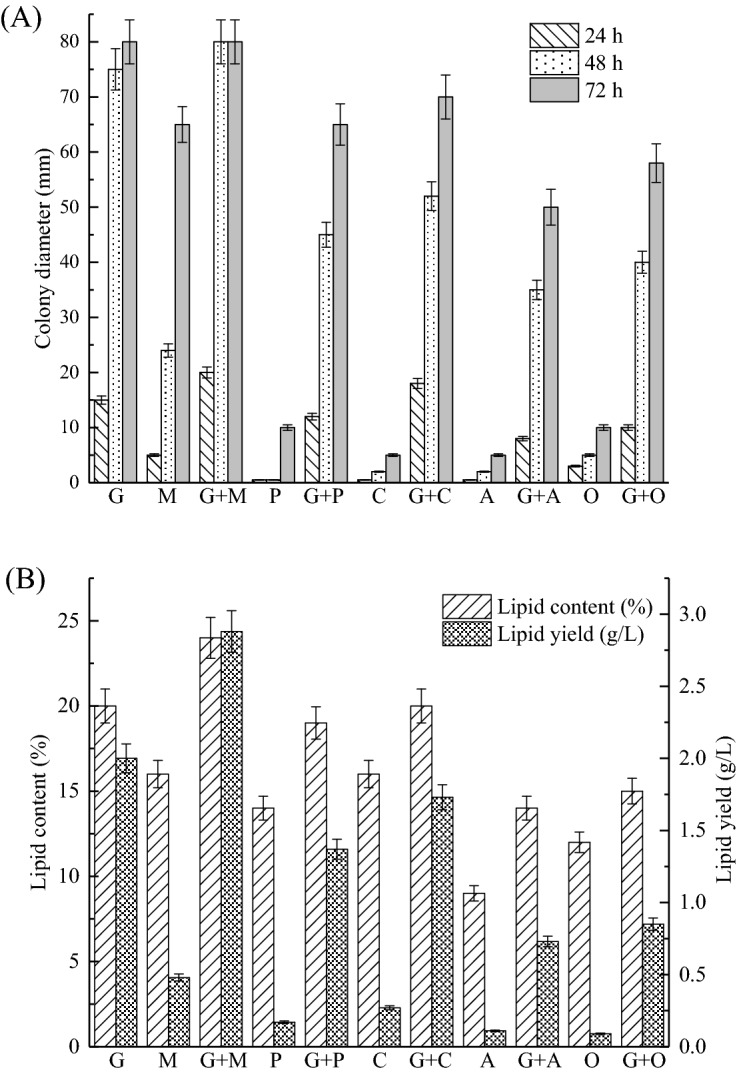
Comparison of cell growth (**A**) and lipid production (**B**) of Mc-MT-2 in batch cultures containing different intermediates. The main carbon source components are abbreviated as follows: G: glucose; M: malic acid; G + M: glucose and malic acid; P: pyruvate; G + P: glucose and pyruvate; C: citric acid; G + C: glucose and citric acid; A: acetic acid; G + A: glucose and acetic acid; O: oxaloacetate; G + O: glucose and oxaloacetate. Values were mean of three independent fermentation experiments. Error bars represent the standard error of the mean.

Maintaining the other nutrients in the medium unchanged, glucose or intermediate metabolites or a complex of glucose and metabolites were used respectively as carbon sources to investigate the effects of different metabolic intermediates on cell growth and lipid production of Mc-MT-2, and results were shown in Fig. 1. It has found from Fig. 1A that, except for malic acid, when other metabolites such as pyruvate, citric acid, acetic acid, oxaloacetic acid, etc. were used as the sole carbon source, the strain basically did not grow. This strain could use malic acid as the sole carbon source for growth, but it does not grow as well as that of glucose. When using a combined carbon source of glucose and intermediate metabolites, the growth of strain is much better than only using the metabolites as the carbon source, but slightly lower than the growth of that using glucose alone.

Changes in lipid production was as similar as the cell growth. From Fig. 1B, when using metabolic intermediates as the sole carbon source, the lipid content and lipid yield of strain were far lower than that of using glucose alone. Except for malic acid, even if a complex of glucose and other metabolites were used, it did not significantly improve the lipid production, which indicated that these TCA metabolic intermediates such as pyruvate, citric acid, acetic acid and oxaloacetic acid could not effectively promote lipid synthesis. However, it is worth noting that when glucose is compounded with malic acid as carbon sources, the lipid production of strain were improved by 1.20-fold for the lipid content and 1.44-fold for the lipid yield compared with that of using glucose alone.

As known, two key factors in lipid biosynthesis are the provision of acetyl-CoA, as the essential precursor of fatty acids, and the provision of NADPH which is consumed in large quantities during the synthesis process^23^. Acetyl-CoA is initially generated from citrate in the mitochondrion, and then translocated by the mitochondrial citrate transport system (citrate/malate/pyruvate transporter proteins) into cytosol in oleaginous fungus *M. circinelloides*^13,24^. The reducing power NADPH for fatty acids synthesis generated in many oleaginous microorganisms by NADP^+^-dependent malic enzyme has been confirmed^25–27^. However, recent studies have found that NADP^+^-dependent malic enzyme are not the only factors that limit lipid accumulation^28^. And Ratledge's chemical calculation analysis also showed that the reducing power NADPH produced by NADP + -ME cannot meet the needs of fatty acid synthesis, and NADP + -ME is not the only provider of NADPH required for lipid synthesis^23^. Moreover, our previous ^13^C metabolic flux analysis confirmed that PP (Pentose Phosphate, PP)pathway might play an important role for supplying NADPH and malic enzyme is not a limiting factor for fatty acid synthesis in oleaginous fungus *M. circinelloides* strains^22,29^. Based on the above researches, it might be inferred that the addition of malic acid could prompt the function of malate transporter in citrate/malate/pyruvate shuttle to deliver acetyl-CoA from the mitochondria to the cytosol^13,15,16^. Moreover, malic acid can be used directly by malic enzyme to generate NADPH and it may also drive PP pathway to supply NADPH^22,29^. Therefore, adding malic acid could regulate the amount of acetyl-CoA and NADPH available for lipid biosynthesis, which led to increased lipid accumulation in the malate transporter overexpressing strain (Mc-MT-2). Consequently, malic acid was chosen for further studies designed to improve the lipid production.

### Effect of malic acid concentration on cell growth and lipid production

**Figure 2:**
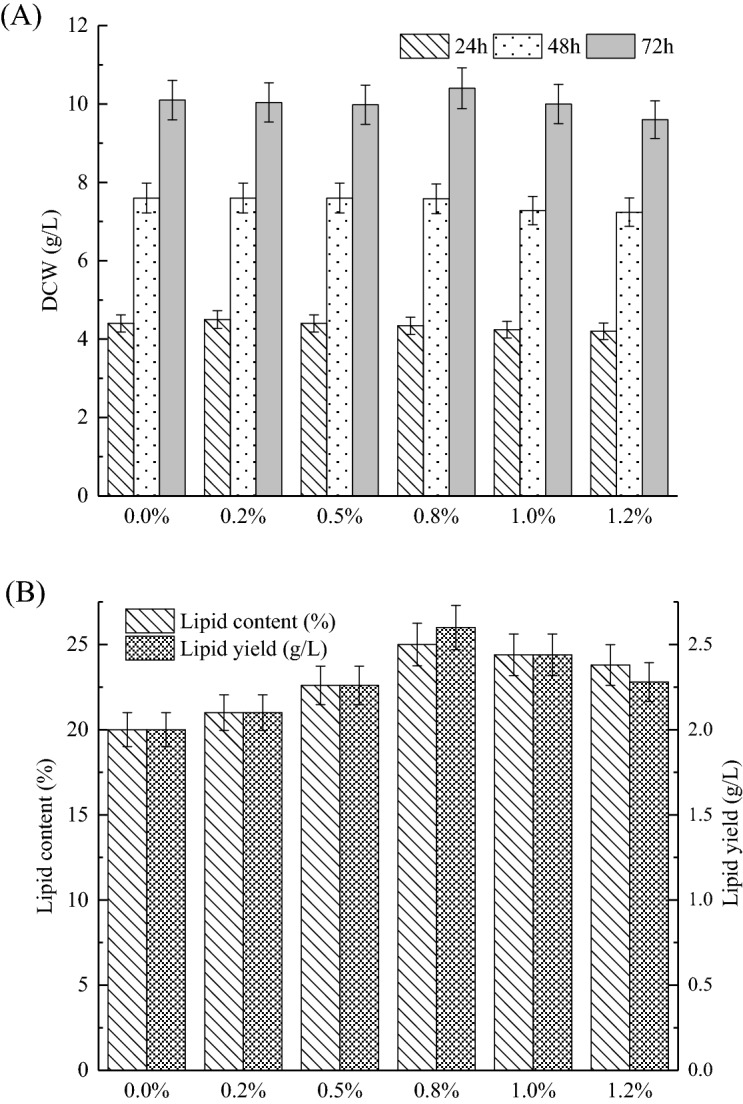
Comparison of cell growth (**A**) and lipid production (**B**) of Mc-MT-2 in batch cultures containing different concentration (0–1.2%, w/w) of malic acid. Values were mean of three independent fermentation experiments. Error bars represent the standard error of the mean.

Although supplementation of the culture medium with malic acid enhanced the lipid production, excessive addition of malic acid might weaken the lipid accumulation and exert negative effects on cell growth^18^. The reason might be that despite having effects on malate-citrate shuttle, malate can also be used directly by malic enzyme to generate NADPH, as well as entering the tricarboxylic acid (TCA) cycle^13,25,27^, however, excessive addition of malic acid will display a decrease in pH of the medium which is not conductive to the cell growth and metabolism^18^. Thus, it is necessary to investigate an optimal malic acid concentration for cell growth and lipid production (as shown in Fig. 2). It was found from Fig. 2A that cell growth was not affected by the addition of malic acid lower than 1%, particularly when the malic acid concentration was higher than 1.2%, cell growth was slightly inhibited. When 0.2–0.8% (w/w) malic acid was added to batch cultures, the lipid production improved remarkably along with the malic acid concentration increased (Fig. 2B). The reason might be that the more malic acid can provide carbon source for the strain, it can also provide more acetyl-CoA and NADPH for lipid synthesis. When the optimal concentration of malic acid (0.8%) was added, the lipid content and lipid yield reached 25% and 2.6 g/L, which were 1.25- and 1.30-fold higher than that of control, respectively. The control was performed in regular nitrogen-limited basic medium without malic acid. However, when the amount of malic acid continued to be increased, the lipid production gradually decreased. Therefore, 0.8% malic acid was chosen for further studies to improve lipid production.

### Effect of the timing of malic acid supplementation on cell growth and lipid production

**Figure 3:**
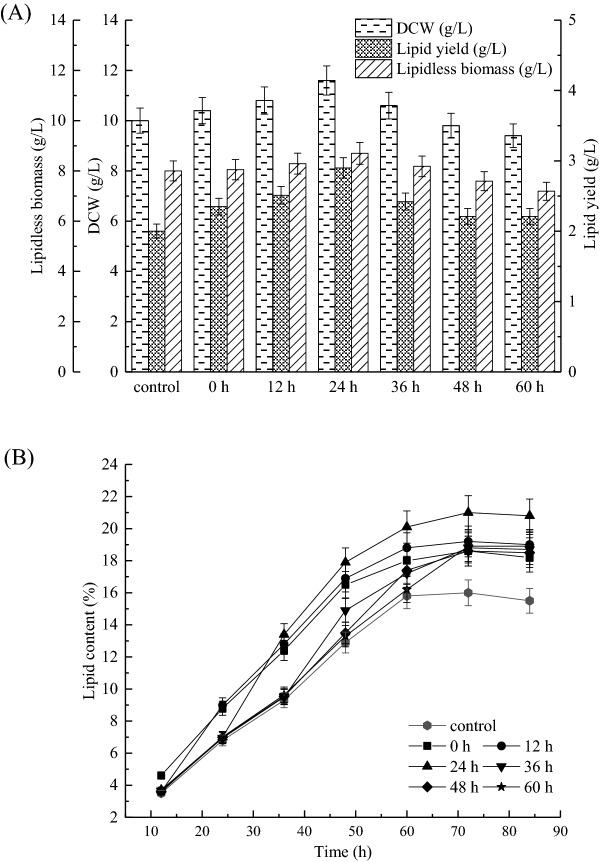
Cell growth and lipid production of Mc-MT-2 as a function of the time when 0.8% malic acid was added to the culture medium. (**A**) Cell growth and lipid yield were measured at the end of batch fermentation when malic acid was added to the culture medium at 0, 12, 24, 36, 48 or 60 h respectively after inoculation. (**B**) Time course of lipid content with the addition of 0.8% malic acid at different times after inoculation. Values were mean of three independent fermentation experiments. Error bars represent the standard error of the mean.

Early research has shown that nitrogen source limitation is an important nutrient feature for oleaginous microorganisms to accumulate lipids. When the nitrogen source is exhausted, the TCA cycle is blocked. At this time, the cells no longer grow, and a large amount of carbon sources continue to be absorbed and converted into lipids^30^. Thus, oleaginous microbial fermentation had experienced two stages of cell growth and lipid synthesis, and the addition point of malic acid might play a prevalent impact on lipid accumulation. In order to explore optimal time for malic acid supplementation, experiments were conducted to add malic acid (0.8%) to the batch cultures at 0, 12, 24, 36, 48 or 60 h, respectively (Fig. 3). It can be seen from the analysis of lipidless biomass (Fig. 3A) that compared with the control, the addition of malic acid in the early stage of fermentation slightly accelerated cell growth, while the addition of malic acid in the middle and late stages of fermentation basically exerted little effect on cell growth. The reason might be that nitrogen was probably limiting after 24 h of fermentation which causes growth to cease, and thus the effect of adding malic acid on the cell growth is not significant in the absence of nitrogen. However, the addition of malic acid significantly increased the lipid yield, especially in the early stage of fermentation. And the increased dry cell biomass (DCW) is mainly due to the increase in lipid quality after the nitrogen source is exhausted (Fig. 3A). On the other hand, the results from Fig. 3B showed that the lipid content gradually enhanced with the extension of fermentation time, and the lipid content was basically stable until fermentation of 60 h. Similar to the growth, the addition of malic acid at early time points has a greater impact on lipid content than adding it in the middle or late stages. Moreover, when malic acid was added at 24 h, the obtained biomass (11.6 g/L), lipid yield (2.9 g/L) and lipid content (25%) reached the highest, which were improved by 1.16-, 1.45- and 1.25-fold compared with the control, respectively. Therefore, addition time of malic acid at 24 h was the optimal condition.

### Development of a two-stage glucose feeding strategy with malic acid supplementation

**Figure 4:**
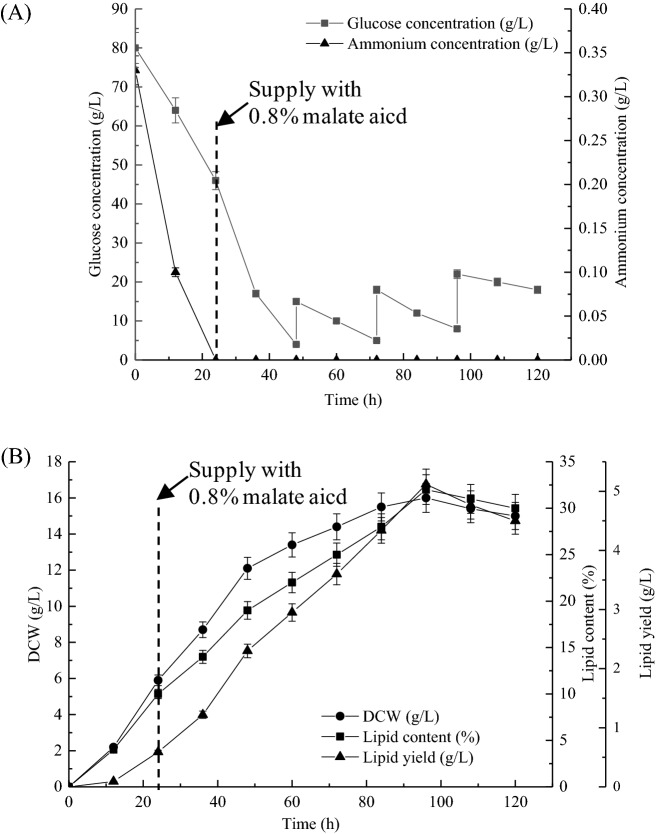
Fed-batch culture of Mc-MT-2 with a glucose feeding strategy and supplementation of malic acid. Residual glucose and ammonium concentration (**A**), cell dry weight, lipid content and lipid yield (**B**) were determined every 12 h in the fermentation process. Values were mean of three independent fermentation experiments. Error bars represent the standard error of the mean.

Early studies have shown that nitrogen limitation is an important nutrient feature for oleaginous microorganisms to accumulate lipids^11,22,30^. The NH_4_^+^ concentration in the culture was detected in Fig. 4A and the result showed that NH_4_^+^ slumped markedly and exhausted within 24 h under limited nitrogen condition. When the nitrogen source in the culture is exhausted, the cells no longer grow at this time, and a large amount of carbon source (such as glucose) in the culture continues to be absorbed and converted into lipids, causing a large accumulation of intracellular lipids^11,30^. However, the primary glucose has been completely consumed in the later stage of above-mentioned batch fermentation, which is not conducive to the accumulation of lipids. In this study, a two-stage glucose feeding strategy was applied to achieve high lipid yield (Fig. 4). After inoculation, the DO value decreased suddenly with a concomitant decrease in pH and glucose. In the early stage of fermentation, the glucose content gradually decreases with the extension of fermentation time (Fig. 4A). At about 48 h, the initial glucose was completely consumed, as indicated by a sudden increase in both dissolved oxygen (DO) and pH, the feeding phase of fed-batch cultivation was initiated. From then on, glucose feeding solutions were added every 24 h to keep the glucose content at about 10–15 g/L in the fermentation broth, which ensured that sufficient carbon source could be continuously converted into lipids.

Furthermore, the results in batch fermentation described above demonstrated that lipid production is obviously enhanced by the addition of malic acid to the growth medium. Thus, fed-batch fermentation strategy was combined with supplement of 0.8% malic acid during fermentation for 24 h to achieve increased lipid accumulation. As depicted in Fig. 4B, the fed-batch processes of cell grow and lipid accumulation were similar, which gradually increased with time during the pre-fermentation stage and reached the highest values at 96 h, and then began to gradually decreased. Noteworthly, it was apparent that the final highest biomass, lipid content and lipid yield reached 16 g/L, 32% and 5.12 g/L in fed-batch fermentation, which was 1.60-, 1.60- and 2.56-fold higher than that of the batch control without supplement of malic acid, respectively. The reason for the obvious improvement in the lipid accumulation might be the combined effect of glucose feeding and addition of malic acid.

Moreover, the fatty acid composition of recombinant *M. circinelloides* Mc-MT-2 in fed-batch culture (Table 1) were basically palmitic acid (C16:0) and octadecenoic acid (C18:1 and C18:2), which were a little different from our previous study^13,22^. It should be noted that the content of oleic acid (C18:1) gradually increased and accumulated with the extension of fermentation, while the content of linolenic acid (C18:3) obviously decreased. This suggested that although the total lipid content is relatively high, a large amount of oleic acid had not been converted into functional polyunsaturated fatty acids in the process of lipid synthesis. Further study for the introduction of modifications to enhance the activities of desaturases would be needed to solve this problem^12^.

**Table 1:** The fatty acid composition of Mc-MT-2 in fed-batch culture.

Time (h)	Fatty acid composition (relative %, w/w of total fatty acids)							
	14:0	16:0	16:1	18:0	18:1	18:2	18:3	Others
12	ND	23.1 ± 1.1	ND	5.5 ± 0.3	26.8 ± 1.0	20.6 ± 0.8	20.1 ± 0.8	3.9 ± 0.2
24	ND	21.2 ± 0.2	ND	9.3 ± 0.3	36.1 ± 0.8	15.7 ± 0.3	13.5 ± 0.6	4.1 ± 0.2
36	ND	22.3 ± 0.8	ND	8.7 ± 0.2	39.7 ± 1.6	13.9 ± 0.6	11.8 ± 0.4	3.7 ± 0.1
48	0.4 ± 0.0	21.6 ± 1.0	1.0 ± 0.0	8.0 ± 0.3	41.2 ± 1.2	13.1 ± 0.4	11.0 ± 0.5	3.7 ± 0.1
60	0.4 ± 0.0	21.3 ± 0.5	1.1 ± 0.0	8.1 ± 0.3	42.9 ± 1.7	12.4 ± 0.2	9.9 ± 0.3	3.8 ± 0.0
72	0.4 ± 0.0	20.9 ± 0.6	1.1 ± 0.0	8.0 ± 0.2	43.7 ± 0.7	12.3 ± 0.3	9.7 ± 0.3	3.9 ± 0.0
84	0.4 ± 0.0	19.7 ± 0.9	1.2 ± 0.0	7.8 ± 0.2	44.7 ± 0.9	12.5 ± 0.5	9.4 ± 0.3	4.4 ± 0.1
96	0.3 ± 0.0	19.8 ± 0.3	1.3 ± 0.0	7.4 ± 0.3	45.3 ± 1.5	12.4 ± 0.3	8.9 ± 0.2	4.4 ± 0.2
108	0.3 ± 0.0	20.1 ± 0.7	1.5 ± 0.0	6.9 ± 0.2	45.7 ± 1.9	12.5 ± 0.8	8.9 ± 0.3	4.1 ± 0.0
120	0.3 ± 0.0	19.6 ± 1.0	1.5 ± 0.0	6.4 ± 0.3	45.8 ± 1.0	12.8 ± 0.5	9.0 ± 0.3	4.7 ± 0.2

Strain was cultured in a 3 L fermenter with a glucose feeding and supplementation of malic acid at 30 °C with stirring at 500 rpm and aeration at 0.5 v/v min^-1^ for 120 h. The fatty acid composition was displayed at different point times. The values are means ± standard deviations of two independent experiments. ND: not detected.

## Conclusion

In this work, an in-depth analysis on supplement of metabolite control strategy to achieve high lipid production was investigated in recombinant *M. circinelloides* Mc-MT-2. We have found that the highest cell growth and lipid production were obtained through a fed-batch culture with glucose feeding and supplementation of 0.8% malic acid at fermentation of 24 h. The final biomass of 16 g/L, a lipid content of 32%, a lipid yield of 5.12 g/L in a controlled bench-top bioreactor, was respectively improved by 1.60-, 1.60- and 2.56-fold compared with the batch control without supplement of malic acid. These findings proves valuable for enhancing microbial lipid production through metabolic supplementation strategy in industrial applications.

## Data Availability

All data generated or analysed during this study are included in this published article.
